# A century of innovation in life sciences at Sun Yat-sen University

**DOI:** 10.1007/s44307-024-00048-2

**Published:** 2024-11-01

**Authors:** Jianhua Yang, Shi Xiao

**Affiliations:** 1 MOE Key Laboratory of Gene Function and Regulation, State Key Laboratory of Biocontrol, School of Life Sciences, Sun Yat-Sen University, Guangzhou, 510275 China; 2 State Key Laboratory of Biocontrol, Guangdong Provincial Key Laboratory of Plant Stress Biology, School of Agriculture and Biotechnology, Sun Yat-Sen University, Shenzhen, 518107 China

The School of Life Sciences (SLS) at Sun Yat-sen University, originally the Department of Biology established in 1924, as one of the earliest biology departments in China. Equipped with excellent teaching and research facilities, SLS explores a broad spectrum of biological research, from traditional to cutting-edge research, encompassing cell and molecular biology, ecological and evolutionary biology, bioinformatics, biotechnology, as well as interdisciplinary investigation in life sciences. The school boasts over 150 faculty, including one Academician of Chinese Academy of Engineering and one Academician of Academia Sinica (Taiwan, China), and excels in global rankings, with several disciplines in the top 1‰ and others in the top 1% according to ESI. SLS hosts more than 10 key research platforms, including State Key Laboratory of Biocontrol, China-ASEAN Belt and Road Joint Laboratory on Marine Aquaculture Technology, and Key Laboratory of Gene Function and Regulation of the Ministry of Education (MOE), ensuring the SLS researchers’ top-quality academic achievements on the world stage. Over the past decades, SLS has trained a large number of excellent alumni, who contribute to the various areas in both academic and non-academic positions all over the world. Situated in Guangdong-Hong Kong-Macao Greater Bay Area, SLS is committed to build a world-class educational and research hubs for life sciences, dedicating to serve the needs of life health and agricultural security in China and the world.

As Sun Yat-sen University’s Biology Department approaches its centennial in November 2024, a special issue will commemorate a century of achievements in the field. We are pleased to invited six outstanding alumni and faculty members of Sun Yat-sen University to present articles selected for publication in this special issue, comprising one original research articles, four reviews and one database article. An overview of the studies included in this issue is provided as follows.

Yongbo Xia and colleagues from Professor Yongchang Cao’s lab at Sun Yat-sen University are well-known for their substantial contributions to animal virology and the control of infectious diseases in livestock. In this special issue, they developed a CRISPR-Cas12a-based assay for detecting swine enteric coronaviruses (SECoVs), offering a rapid, sensitive, and specific diagnostic tool. The study demonstrated high agreement with RT-qPCR, highlighting the assay's potential for field application in monitoring SECoVs, crucial for swine industry and public health (Xia et al. 2024).

Bin Li and colleagues from Professor Liang-Hu Qu’s lab at Sun Yat-sen University have developed various computational and experimental methods to discover novel classes of non-coding RNAs (ncRNAs) and their functions and mechanisms, achieving remarkable progress in RNA research (Li et al. 2024a; Liu et al. 2024). In this special issue, the authors explored the complex role of oncogenes in inducing cellular plasticity, which significantly impacts tumor therapy. Their review emphasized the paradoxical effects of oncogenes in promoting cell proliferation while inhibiting metastasis, underscoring the need for precision targeting strategies to effectively combat cancer without exacerbating metastatic potential. This study provided valuable insights into the multifaceted nature of oncogene-induced cellular plasticity and its implications for cancer treatment (Li et al. 2024b).

Jian-Hua Zhao and colleagues from Professor Hui-Shan Guo’s team at the Chinese Academy of Sciences have made significant contributions to the fields of RNA silencing and plant disease resistance. In this special issue, they examined the challenges and potential of RNAi-based strategies for crop protection, including HIGS, SIGS, and MIGS. They emphasized the importance of dsRNA design, production, and delivery, and speculated on the inheritance of RNAi effects across generations. This review underscored the need to address these challenges to enhance sustainable agriculture through RNAi technology (Zhao et al. 2024).

Jia-Rui Han and colleagues from Professor Wenjun Li’s team at Sun Yat-sen University have made significant contributions to the fields of microbial systematics and ecology, particularly in the discovery of new bacterial and archaeal taxa and the exploration of microbial diversity in extreme environments. In this special issue, they provided a comprehensive roadmap for mining microbial and metabolic dark matter in extreme environments. They underscored the significance of multi-omics data in advancing our understanding of extremophiles' diversity, ecological roles, and biosynthetic potential, offering valuable insights for discovering novel natural products and enhancing microbial ecology and evolution knowledge (Han et al. 2024).

Chenqiu Zhang and colleagues from Professor Jun Cui’s lab at Sun Yat-sen University have made significant contributions to the understanding of innate immune recognition and regulation. In this special issue, they developed DSCI, a comprehensive database for synthetic biology components in innate immunity, facilitating cell engineering decision-making. The study meticulously curated over 4000 entries, offering insights into signaling pathways and motifs, and supporting gene circuit construction strategies. This work underscored the importance of specialized databases in advancing medical synthetic biology and immunotherapy (Zhang et al. 2024).

Wenteng Xu and colleagues from Professor Songlin Chen's team are advancing fish breeding into the genomic era, making significant contributions that place China's aquatic biotechnology at the forefront globally. In this special issue, they review the pivotal role of biotechnology in enhancing China’s marine aquaculture, focusing on genomic innovations and breeding strategies. They highlighted significant progress in genome sequencing, sex-related marker screening, genomic selection, and genome editing, which were revolutionizing marine fish breeding. The study underscored the potential of these technologies in addressing food security and enhancing the sustainability of aquaculture practices (Xu et al. 2024).

Over the past few decades, the field of biotechnology has witnessed remarkable progress, encompassing a wide range of advancements in molecular biology and genetic engineering, which have been driven by innovative experiments and sophisticated algorithms (Huang et al. 2023). These six articles in this special issue collectively broaden our understanding of cutting-edge biotechnological advancements. They elucidate the roles of innovative assays, oncogene-induced cellular plasticity, RNAi-based crop protection, multi-omics in extremophile research, synthetic biology databases, and genomics in marine aquaculture. We envision that new breakthroughs in these areas will underscore the complexity and importance of biotechnology in determining the future of agriculture, healthcare, and environmental sustainability, hinting at potential novel applications in diagnostics, cancer therapy, sustainable farming, microbial ecology, synthetic biology, and aquaculture practices.
